# A comparative study of advanced evolutionary algorithms for optimizing microgrid performance under dynamic pricing conditions

**DOI:** 10.1038/s41598-024-54829-9

**Published:** 2024-02-24

**Authors:** Rasha Elazab, Ahmed T. Abdelnaby, A.A. Ali

**Affiliations:** Faculty of Engineering, Helwan University, Cairo, Egypt

**Keywords:** Electrical and electronic engineering, Energy grids and networks, Power distribution, Power stations, Batteries, Solar energy, Wind energy

## Abstract

The integration of microgrids into the existing power system framework enhances the reliability and efficiency of the utility grid. This manuscript presents an innovative mathematical paradigm designed for the optimization of both the structural and operational aspects of a grid-connected microgrid, leveraging the principles of Demand-Side Management (DSM). The focus of this work lies in a comprehensive exploration of the implications brought about by the Renewable Generation-Based Dynamic Pricing Demand Response (RGDP-DR) mechanism, particularly in terms of its influence on the optimal microgrid configuration, considering perspectives from end-users and the utility entity. This inquiry is rooted in a holistic assessment that encompasses technical and economic performance benchmarks. The RGDP-induced DR framework adeptly addresses the needs of the consumer base, showcasing notable efficiency and economic feasibility. To address the intricate nonlinear optimization challenge at hand, we employ an evolutionary algorithm named the "Dandelion Algorithm" (DA). A rigorous comparative study is conducted to evaluate the efficacy of four optimization techniques, affirming the supremacy of the proposed DA. Within this discourse, the complexity of microgrid sizing is cast as a dual-objective optimization task. The twin objectives involve minimizing the aggregate annual outlay and reducing emissions. The results of this endeavor unequivocally endorse the superiority of the DA over its counterparts. The DA demonstrates exceptional proficiency in orchestrating the most cost-effective microgrid and consumer invoice, surpassing the performance of alternative optimization methodologies.

## Introduction

In contemporary energy landscapes, there has been a noteworthy upswing in the integration of Renewable Energy Sources (RES) with the grid, driven by a commitment to reduce greenhouse gas emissions from conventional fossil fuel-based power plants^1^. This transformative era has witnessed the ascendancy of decentralized RES, strategically positioned to optimize clean energy generation across extensive geographical domains, culminating in the emergence of Microgrid (MG) concepts as compact-scale networks^2^.

Energizing the paradigm of Demand-Side Management (DSM), this paper underscores the imperative for customers to manipulate their energy consumption patterns. The effective application of DSM brings multifaceted benefits, including enhanced system reliability, improved efficiency, reduced microgrid operational costs, optimized load patterns, minimized power outages, decreased carbon emissions, and increased customer satisfaction. Within Demand Response (DR), specifically, load modification strategies, a subset of DSM, various tactics such as strategic load growth, load shifting, valley filling, peak clipping, strategic conservation, and flexible load shaping are employed^3^. These strategies fall into two main approaches: incentive-based and price-based paradigms^4^. The former encompasses initiatives such as demand buyback/bidding schemes^5^, curtailable /interruptible services^6^, ancillary service programs^7^, emergency DR programs^8^, capacity market programs^9^, and direct load control mechanisms^10^. Conversely, the latter embraces strategies: Time-of-Use (TOU), fixed pricing, real-time pricing, and critical peak pricing models^11^.

In the microgrid planning scope, two pivotal factors—the operator perspective and the client outlook—significantly influence the effectiveness of DSM deployment. From the client's standpoint, electricity bill expenditure is ameliorated, while on the operator's side, DSM engenders a reduction in microgrid overheads and mitigates risks entailed by power deficits^12^. The literature summarizes an array of techniques and mathematical formulations underpinning DSM within MG applications^13^, with a notable exploration into the comparative assessment of diverse Energy Storage Systems (ESS) for DSM through industrial installations detailed in^14^.

However, the intricate challenge of microgrid sizing, entangled with non-linear constraints, necessitates the integration of DR programs. Recent initiatives explored in references^15–17^ investigated Incentive-Based Demand Response Programs (IDRPs) employing the Sparrow Algorithm, Black Widow Algorithm (BWA), and Whale Algorithm to reduce operational expenses. The practice of load shifting was examined using Mixed-Integer Linear Programming (MILP), Genetic Algorithm (GA), and Augmented ε-Constraint techniques to optimize overall expenditures^18–20^. TOU strategies are featured with BWA, GA, hybrid optimization multi-energy resource, and pseudo-gravitational algorithm to drive cost reduction^21–24^.

In^25–28^, the optimal sizing of various microgrids considering energy management techniques using several optimization algorithms has been discussed.

In 2020, Renewable Generation-Based Dynamic Pricing (RGDP) DR was proposed in^29^ to minimize the total MG cost of an isolated microgrid using MILP. RGDP-DR achieves a zero reduction in energy consumption and maximum customer satisfaction. However, this paper adopts RGDP DR to minimize life cycle emissions and the overall cost of grid-tied MG using the Dandelion Algorithm (DA).

Given the aforementioned investigations, the commonality across prior studies is the trade-off between DR-driven energy reduction and customer satisfaction. This research addresses this gap by introducing a novel DR strategy termed RGDP DR, designed for rescheduling load demands within grid-connected MGs while prioritizing customer satisfaction. To effectively handle this intricate challenge, a novel meta-heuristic approach called the Dandelion Algorithm (DA) is proposed. The primary objective of this algorithm is to determine optimal capacities for distributed energy sources within the microgrid, taking into account the complexities of DSM. A comprehensive comparative analysis is undertaken, comparing the performance of the DA against three alternative optimization methods within the context of grid-connected MGs influenced by the RGDP DR strategy. Through the utilization of MATLAB/M-files simulation software, a mathematical model of the grid-connected MG is established, incorporating the RGDP DR strategy and various optimization techniques. This model serves to demonstrate the effectiveness of the proposed approach in contrast to its counterparts.

Moreover, the modifications introduced in our study are crucial for the specific context of grid-connected microgrids. While the original mathematical model^29^ was formulated for an isolated microgrid, we have diligently adapted and validated the model to align with the distinctive characteristics of a grid-tied microgrid. This adaptation encompasses the inclusion of considerations for energy exchange with the utility grid—a facet not explicitly addressed in earlier literature focused on isolated microgrids. The cost functions have been appropriately modified to account for this interaction with the utility grid. More precisely, we have incorporated the price of energy exchanged with the utility grid into the cost functions. This refinement ensures a more precise representation of the economic dynamics and operational constraints inherent in grid-connected microgrids. By doing so, our study provides a comprehensive perspective on the optimization challenges and opportunities specifically applicable to the context of grid-connected microgrids.

The prime contributions of this study are concisely summarized as follows:

- Pioneering the integration of a groundbreaking price-based DR paradigm, namely RGDP DR, designed to ensure maximal customer contentment at a reduced operational outlay, within the context of grid-connected MGs.
- Developing an innovative mathematical framework that seamlessly integrates the Demand Response (DR) approach into the optimization challenge of identifying the most efficient dimensions for grid-connected microgrids. This framework aims to achieve two simultaneous goals: the reduction of overall costs and the mitigation of emissions.
- Introducing a cutting-edge metaheuristic algorithm, DA, specifically designed to adeptly address the complexities associated with optimizing the size of grid-connected microgrids. The algorithm capitalizes on technical and economic metrics to effectively navigate the inherent intricacies of this optimization problem.

The subsequent sections of the paper are systematically organized as follows: Section “Modeling and configuration of system components” provides a concise explanation of the configuration and modeling complexities related to the proposed grid-connected MG. Section “Problem formulation” presents the formulation of the optimization challenge, incorporating the system's inherent constraints. In Section “RGDP-DR program”, a comprehensive depiction of the RGDP DR strategy is provided, along with its intricate modeling. The methodologies underlying the utilization of four distinct optimization techniques to address the optimization problem are concisely summarized in Section “Optimization techniques”. Moving forward, Section “Results and discussion” serves as a platform for a thorough examination and discussion of the simulation outcomes across four distinct scenarios. Bringing the discussion to a close, Section “Conclusion” summarizes the cumulative conclusion of this study.

## Modeling and configuration of system components

**Figure 1:**
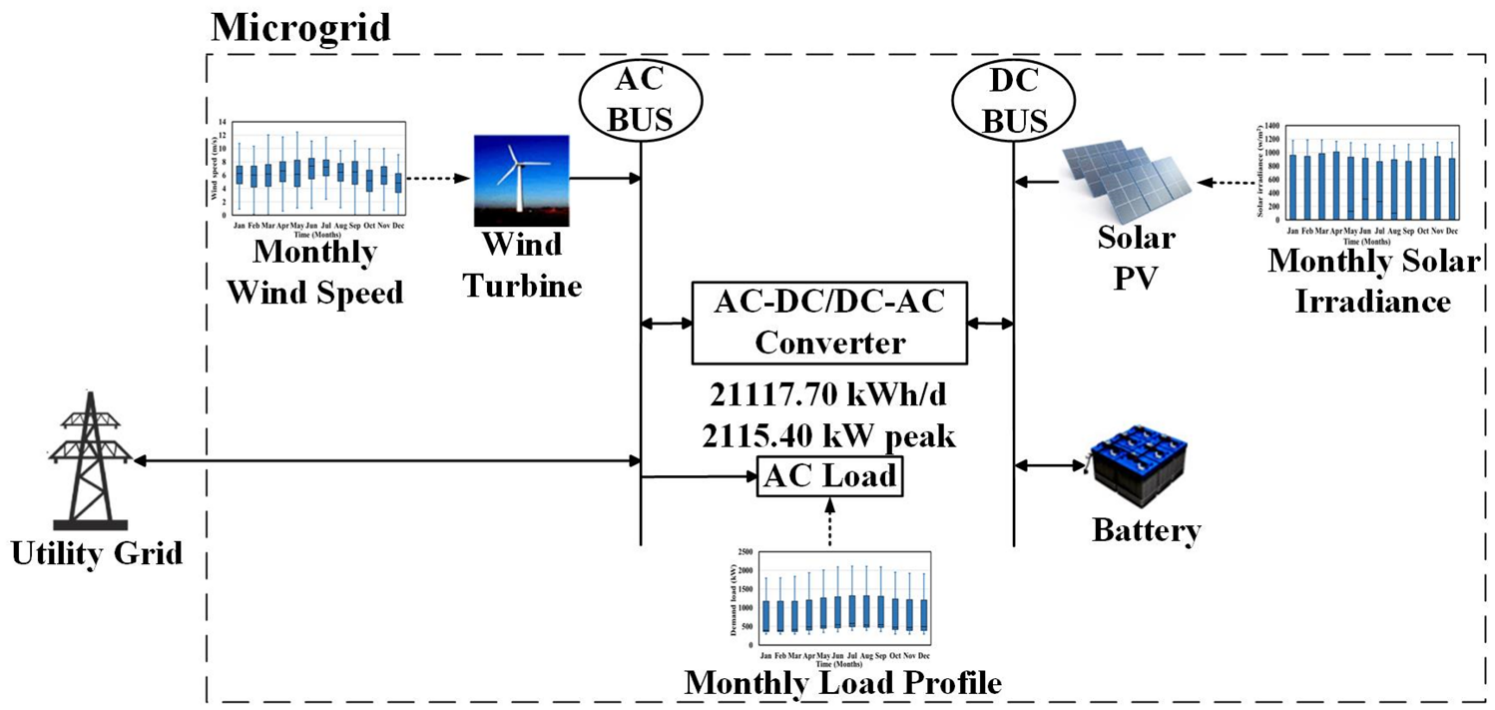
The studied grid-tied MG.

Figure 1 illustrates the arrangement of the proposed MG as described. This MG design incorporates a trio of RES: photovoltaic (PV) panels, battery storage units, and Wind Turbines (WTs). Additionally, a converter is incorporated into the setup to establish a connection between the AC and DC buses. On a specified day, MG registers a peak demand of 2115.4 kW, with a corresponding energy consumption of roughly 21,117.7 kWh. The detailed model for each RES will be discussed in the following subsections:

### PV modeling

The PV-generated power ${P}_{S}\left(t\right)$ can be computed using Eq. (1) ^30^.

1 $${P}_{S}\left(t\right)={N}_{S}\times {P}_{STC}\times {F}_{S}\times \frac{I\left(t\right)}{1000}$$

where ${N}_{S}$ is the number of PV modules, ${P}_{STC}$ is the PV power rating at STC (kW), ${F}_{S}$ is the PV module reduction factor, and $I\left(t\right)$ is the global solar irradiance (W/m^2^).

### WT modeling

WT output power ${P}_{w}\left(t\right)$ can be defined using Eq. (2)^31^.

2 $${P}_{w}\left(t\right)=\left\{\begin{array}{l} 0\, 0\le v\left(t\right)\le {v}_{ci} \\ {N}_{w}\times {P}_{r}\times \frac{{v}^{2}\left(t\right)-{v}_{ci}^{2}}{{v}_{r}^{2}-{v}_{ci}^{2}} {v}_{ci}\le v\left(t\right)\le {v}_{r}\\ {{N}_{w}\times P}_{r} {v}_{r}\le v\left(t\right)\le {v}_{co}\\ 0\, v\left(t\right)\ge {v}_{co}\end{array}\right.$$

where ${P}_{r}$ is the WT’s rated power, and ${N}_{w}$ is the number of WTs.

### Modeling of the battery energy storage system

Recently, the utilization of lithium-ion batteries has become prevalent in MG applications due to their remarkable characteristics, including high power density, significant energy density, and prolonged lifespan. Battery Energy Storage Systems (BESS) function through three distinct operational modes: charging, discharging, and idle mode.

#### Charging mode

In instances where the power generated by MG sources exceeds load requirements, the excess power is directed toward charging the battery. Consequently, the methodology outlined below is employed to calculate the accumulated energy from this charging process^32,33^:

3 $${P}_{CH}\left(t\right)=\left({P}_{S}\left(t\right)+{\eta }_{CON}\times ({P}_{W}\left(t\right)-{P}_{L}^{Z}\left(t\right))\right)\times {\eta }_{CH}$$

where ${P}_{CH}\left(t\right)$ is the power being charged at time $t$, ${P}_{L}^{Z}\left(t\right)$ signifies the load power of the scenario indexed as the ${z}{\text{th}}$ at time $t$, and subscript $z$ indicates the specific scenarios under consideration. ${E}_{CH}\left(t\right)$ represents the energy being charged during the time interval $\Delta t$, which is typically an hour. The efficiencies of the converter and charging processes are denoted as ${\eta }_{CON}$ and ${\eta }_{CH}$, respectively.

The battery State of Charge ($SOC$) is calculated as:

4 $$SOC\left(t\right)=SOC\left(t-1\right)+{P}_{CH}\left(t\right)\times \Delta t$$

where $SOC\left(t\right)$ and $SOC\left(t-1\right)$ are states of charge at two successive time instants of $t$ and $t-1$, respectively.

When the calculated ${P}_{CH}\left(t\right)$ is greater than (${SOC}_{max}$ − $SOC\left(t-1\right)$), the power can be sold to the connected grid ${P}_{GS}\left(t\right)$ and calculated as:

5 $${P}_{GS}\left(t\right)\times \Delta t={P}_{CH}\left(t\right)\times \Delta t-{SOC}_{max}+SOC\left(t-1\right)$$

where ${SOC}_{max}$ refers to the maximum SOC of the battery.

#### Discharging mode

In cases where the energy demand of a load exceeds the output power of PV and WTs, the battery is discharged. The subsequent energy discharged and the corresponding SOC can be approximated using the following approach^32,33^:

6 $${P}_{DIS}\left(t\right)=\frac{\frac{{{P}_{L}^{Z}\left(t\right)-P}_{W}\left(t\right)}{{\eta }_{CON}}-{P}_{S}\left(t\right)}{{\eta }_{DIS}}$$

7 $$SOC\left(t\right)=SOC\left(t-1\right)-{P}_{DIS}\left(t\right)\times \Delta t$$

where ${P}_{DIS}\left(t\right)$ is battery discharging power at time $t$, ${E}_{DIS}\left(t\right)$ stands for discharging energy. ${\eta }_{DIS}$ is the discharging efficiency.

In the event of a battery power shortage, the purchased grid power ${P}_{GP}\left(t\right)$ can be defined as:

8 $${P}_{GP}\left(t\right)\times \Delta t={P}_{DIS}\left(t\right)\times \Delta t-SOC\left(t-1\right)+{SOC}_{min}$$

where ${SOC}_{min}$ is the battery minimum permissible SOC.

#### Ideal mode

Upon reaching full charge, the surplus MG-generated power can be sold back to the grid. This situation is depicted as follows^34^:

9 $${P}_{GS}\left(t\right)={P}_{W}\left(t\right)-{P}_{L}^{Z}\left(t\right)+{P}_{S}\left(t\right)\times {\eta }_{CON}$$

Once the battery's maximum discharge capacity is reached, any additional power needed will be procured from the grid. This scenario can be articulated as follows:

10 $${P}_{GP}\left(t\right)={P}_{L}^{Z}\left(t\right)-{P}_{W}\left(t\right)-{P}_{S}\left(t\right)\times {\eta }_{CON}$$

## Problem formulation

This section outlines the technical constraints on the system as well as the optimization problem's objective functions.

### The objective functions (OFs)

The study addresses the comprehensive OF inherent in the optimization challenge of microgrid (MG) sizing. The primary objective of this Objective Function (OF) is to simultaneously minimize the Total Annual Cost (TAC) as presented in Eq. (12) and reduce Life Cycle Emissions (LCE) as defined in Eq. (17) within the context of grid-interconnected MG. The optimization process considers operational constraints delineated in Eqs. (18) to (24). Consequently, the overarching objective function is composed of two metrics, each of which is characterized by a specific weight ratio. Therefore, the formulation of the OF is presented as follows:

11 $${{\text{min}}}_{x}({\text{OF}})={{\text{min}}}_{x} \bigg({\mathrm{\varphi }}_{1}*{\text{TAC}}+{\mathrm{\varphi }}_{2}*\frac{{{\text{E}}}_{pen}*{\text{LCE}}}{1000} \bigg)$$

where $$x$$ represents a vector containing optimization parameters: the power output of sources, storage, and converter. $${{\text{E}}}_{pen}$$ stands for the penalty associated with CO_2_ emissions ($/ton). The assigned values for $${\mathrm{\varphi }}_{1}$$ and $${\mathrm{\varphi }}_{2}$$ are both set to 0.5.

#### TAC minimization

It can be calculated as follows^29^:

12 $$TAC={CC}_{AN}+{RPC}_{AN}+{OMC}_{AN}-{SVC}_{AN}$$

where ${CC}_{AN}$, ${RPC}_{AN}$, ${OMC}_{AN}$, and ${SVC}_{AN}$ are MG annual capital costs of the MG components, replacement costs, operating and maintenance costs, and salvage costs, respectively.

##### Components’ capital costs

The total yearly MG components' capital cost can be determined using Eq. (13)^35^:

13 $${CC}_{AN}=\sum_{k=1}^{K}\left\{{N}_{k}\times {P}_{R\_{\text{k}}}\times {C}_{i\_k}* \frac{{D}_{r}{\left(1+{D}_{r}\right)}^{T}}{{\left(1+{D}_{r}\right)}^{T}-1}\right\}$$

where $${N}_{k}$$ indicates the number of $${k}{\text{th}}$$ component units, $${P}_{R\_{\text{k}}}$$ is the capacity in kW of $${k}{\text{th}}$$ component, $${C}_{i\_k}$$ is the $${k}{\text{th}}$$ component initial cost in ($/kW), $$K$$ is the number of system components,$${D}_{r}$$ indicates the reduction rate (%), $$T$$ is the project's lifetime, and $$k$$ indicates the MG components, which are WT, BESS, PV, and converter.

##### Replacement costs

If the lifetimes of microgrid (MG) components are shorter than the lifespan of the project, they need to be replaced. The overall annual cost of replacing MG components can be calculated using Eq. (14)^36^:

14 $${RPC}_{AN}=\sum_{k=1}^{K}\left\{{N}_{k}\times {P}_{R\_{\text{k}}}\times {C}_{r\_k}\times \sum_{m=1}^{{NR}_{k}}{\left(\frac{1}{1+{D}_{r}}\right)}^{\left(m\times {L}_{k}\right)}\right\}*\frac{{D}_{r}{\left(1+{D}_{r}\right)}^{T}}{{\left(1+{D}_{r}\right)}^{T}-1}$$

where $${NR}_{k}$$ indicates the number of $${k}{\text{th}}$$ component replacements, $${L}_{k}$$ is the lifetime in years, and $${C}_{r\_k}$$ is the cost of unit replacement ($/kW).

##### Operating and maintenance O&M costs

Annual O&M costs can be described as^29,30^:

15 $${OMC}_{AN}=\sum_{k=1}^{K}\left\{{N}_{k}*{P}_{R\_{\text{k}}}*{C}_{om\_k}\right\}+\sum_{t=1}^{8760}\left\{{C}_{gp}\left(t\right)*{P}_{gp}\left(t\right)-{C}_{gs}\left(t\right)*{P}_{gs}\left(t\right)\right\}$$

where $${C}_{om\_k}$$ is the $${k}{\text{th}}$$ component annual O&M costs in $/kW/Year, $${C}_{gp}\left(t\right)$$ is the per-unit buying grid power cost at $${t}{\text{th}}$$ hour in $/kW/Year, $${P}_{gp}\left(t\right)$$ (kW) is the purchased power from the utility during the $${t}{\text{th}}$$ hour, $${C}_{gs}\left(t\right)$$ is the price of the grid sold power in $/kW/Year, and $${P}_{gs}\left(t\right)$$ (kW) is the utility sold power.

##### Salvage costs

The annual MG components’ salvage cost can be written as^37^:

16 $${SVC}_{AN}=\sum_{k=1}^{K}\left\{{N}_{k}*{P}_{R\_k}*{C}_{r\_k}*\frac{{L}_{k}-\left(T-\left({L}_{k}*{NR}_{k}\right)\right)}{{L}_{k}}* {\left(\frac{1}{1+{D}_{r}}\right)}^{T}\right\}* \frac{{D}_{r}{\left(1+{D}_{r}\right)}^{T}}{{\left(1+{D}_{r}\right)}^{T}-1}$$

#### Minimizing LCE

${\text{LCE}}$ In kilograms of CO_2_-equivalent per year represents the cumulative carbon dioxide (CO_2_) emissions from microgrid components over their complete lifecycle. This value can be computed using the formula outlined in reference^32^:

17 $${\text{LCE}}=\sum_{k=1}^{K}{F}_{k}{E}_{k}$$

where ${F}_{k}$ is the ${k}{\text{th}}$ component annual CO_2_ emissions in kgCO_2_-eq/kWh and ${{\text{E}}}_{{\text{k}}}$ is the annual generated energy in kWh. MG's detailed characteristics are presented^34^.

Operational parameters of MG components must adhere to established limits to ensure the integrity and dependability of the power supply infrastructure.

### The technical constraints

Operational parameters of MG components must adhere to established limits to ensure the integrity and dependability of the power supply infrastructure. Inequality and power balance constraints should be calculated and considered, as will be described in the following paragraphs.

#### The inequality constraints

Maximum and minimum boundaries of MG sources should be satisfied, as follows^34^:

18 $$0\le {P}_{S}\left(t\right)\le {P}_{S\_max}$$

19 $$0\le {P}_{w}\left(t\right)\le {P}_{w\_max}$$

20 $${-P}_{CH\_max}\le {P}_{b}\left(t\right)\le {P}_{DIS\_max}$$

21 $$0\le \left|{P}_{INV}\left(t\right)\right|\le {P}_{inv\_max}$$

where ${P}_{b}\left(t\right)$ is storage power. ${P}_{S\_max}$, ${P}_{w\_max}$, and ${P}_{inv\_max}$ are RESs and converter capacities, respectively. ${P}_{CH\_max}$ and ${P}_{DIS\_max}$ represent the maximum battery charging and maximum battery discharging, respectively.

The SOC of the battery must be maintained within the acceptable range, as deep discharges and overcharging can both lead to a reduction in battery lifespan, as explained below^32,33^:

22 $${SOC}_{min}\le SOC\left(t\right)\le {SOC}_{max}$$

At time $i$, the RGDP electrical cost ${\rho }_{rgdp}\left(i\right)$ must be within the allowable limits as follows^29^:

23 $${\rho }_{min}\le {\rho }_{rgdp}\left(i\right)\le {\rho }_{max}$$

where ${\rho }_{min}$ and ${\rho }_{max}$ are the minimum and maximum prices, respectively.

#### The power balance constraints

Power equilibrium can be achieved through the utilization of the subsequent expression^34^:

24 $${P}_{L}^{Z}\left(t\right)={P}_{GP}\left(t\right)-{P}_{GS}\left(t\right)+{P}_{W}\left(t\right)+\left({P}_{S}\left(t\right)+{P}_{DIS}\left(t\right)\times {\eta }_{DIS}-\frac{{P}_{CH}\left(t\right)}{{\eta }_{CH}}\right)\times {\eta }_{CON}$$

## RGDP-DR program

An energy management strategy that enables the modification of load patterns is known as DR, where electricity consumption is modified by shifting it from high-demand to low-demand periods or by reducing usage during peak periods. Conventional DR approaches often lead to reduced energy consumption, though they might impact customer satisfaction. On the contrary, the RGDP-DR program distinguishes itself by achieving optimal equilibrium. This program guarantees no reduction in energy consumption, thereby achieving the utmost customer satisfaction, as expounded in this section.

The self-elasticity coefficient $E\left(i,i\right)$ refers to the sensitivity of $\left(i\right)$ hour demand to $\left(i\right)$ hour price and can be expressed using Eq. (25) as follows^29^:

25 $$E\left(i,i\right)=\frac{{\rho }_{o}\left(i\right)}{{P}_{L}\left(i\right)}\times \frac{\partial {P}_{L}\left(i\right)}{\partial \rho \left(i\right)} i=\mathrm{1,2},\dots ,24$$

where ${\rho }_{o}\left(i\right)$ is the initial electrical cost, ${P}_{L}\left(i\right)$ is the initial demand, $\partial {P}_{L}\left(i\right)$ represents the demand variance during period $i$, and $\partial \rho \left(i\right)$ indicates the cost fluctuation during the *i* period.

The cross-elasticity coefficient $E\left(i,j\right)$ reflects the $\left(i\right)$ demand sensitivity to $\left(j\right)$ price and is calculated using Eq. (26) as follows^29^:

26 $$E\left(i,j\right)=\frac{{\rho }_{o}\left(j\right)}{{P}_{L}\left(i\right)}\times \frac{\partial {P}_{L}\left(i\right)}{\partial \rho \left(j\right)} i,j=\mathrm{1,2},\dots ,24$$

Table 1 shows the applied cross- and self-elasticities of the demand load in this research.

**Table 1:** Cross and self-elasticity coefficients^38^.

	Peak	Off-peak	Valley
Peak	− 0.100	0.0160	0.0120
Off-peak	0.0160	− 0.100	0.010
Valley	0.0120	0.010	− 0.100

RGDP signifies an advanced evolution of time-based Demand Response (DR) programs. This advancement arises from the shift in the DR electricity price, moving from a variable independent of microgrid (MG) configuration to a dynamic value linked to the disparity between energy demand and the Renewable Energy Source (RES) output. Furthermore, RGDP's primary objective is to mitigate customer dissatisfaction while simultaneously enhancing utility for the electricity provider.

Consequently, the price structure offered motivates participants to simply reschedule energy usage patterns. As specified in Eq. (27), the comprehensive energy consumption of a participant should remain comparatively consistent both before and after the RGDP DR program's implementation^29^.

27 $$\sum_{i=1}^{24}{P}_{L}^{RGDP}\left(i\right)=\sum_{i=1}^{24}{P}_{L}\left(i\right)$$

The RGDP tariff is quantified as follows^29^:

28 $${\rho }_{rgdp}\left(i\right)={\rho }_{0}\left(i\right)\times \left\{1+\frac{{P}_{L}\left(i\right)-{P}_{W}\left(i\right)-{P}_{S}\left(i\right)\times {\eta }_{CON}}{{P}_{L}\left(i\right)}\right\}$$

The RGDP economic load model is acquired by^29^:

29 $${P}_{L}^{RGDP}\left(i\right)={P}_{L}\left(i\right)\times \left\{1+E\left(i,i\right)\times \frac{\left[{\rho }_{rgdp}\left(i\right)-{\rho }_{0}\left(i\right)\right]}{{\rho }_{0}\left(i\right)}+\sum_{\begin{array}{c}j=1\\ j\ne i\end{array}}^{24}E\left(i,j\right)\times \frac{\left[{\rho }_{rgdp}\left(j\right)-{\rho }_{0}\left(j\right)\right]}{{\rho }_{0}\left(j\right)}\right\}$$

## Optimization techniques

Four optimization techniques applied in this paper are mathematically modelled in this section.

### PSO algorithm

Eberhart and Kennedy first presented PSO^39^. This algorithm draws inspiration from the flocking behavior of birds aiming to reach a target, with each individual's actions influencing the group's collective movement. In PSO, particles symbolize potential solutions dispersed within the search space to address a given problem. The PSO methodology encompasses five fundamental stages:

- *Initialization* The initial setup of particles and their attributes.
- *Evaluation* The assessment of each particle's fitness based on the defined objective function.
- *Updating Particle Best (Pbest)* Individual particles update their personal best solution based on their current fitness.
- *Updating Global Best (Gbest)* The best solution among all particles is updated.
- *Updating Velocity and Location* Particles adjust their velocity and position, guided by both Pbest and Gbest, iteratively moving towards a globally optimal solution.

Particles in PSO emulate the trajectories set by Pbest and Gbest, dynamically altering their directions to progressively converge towards the problem's global optimal solution.

### Kepler optimization algorithm (KOA)

**Figure 2:**
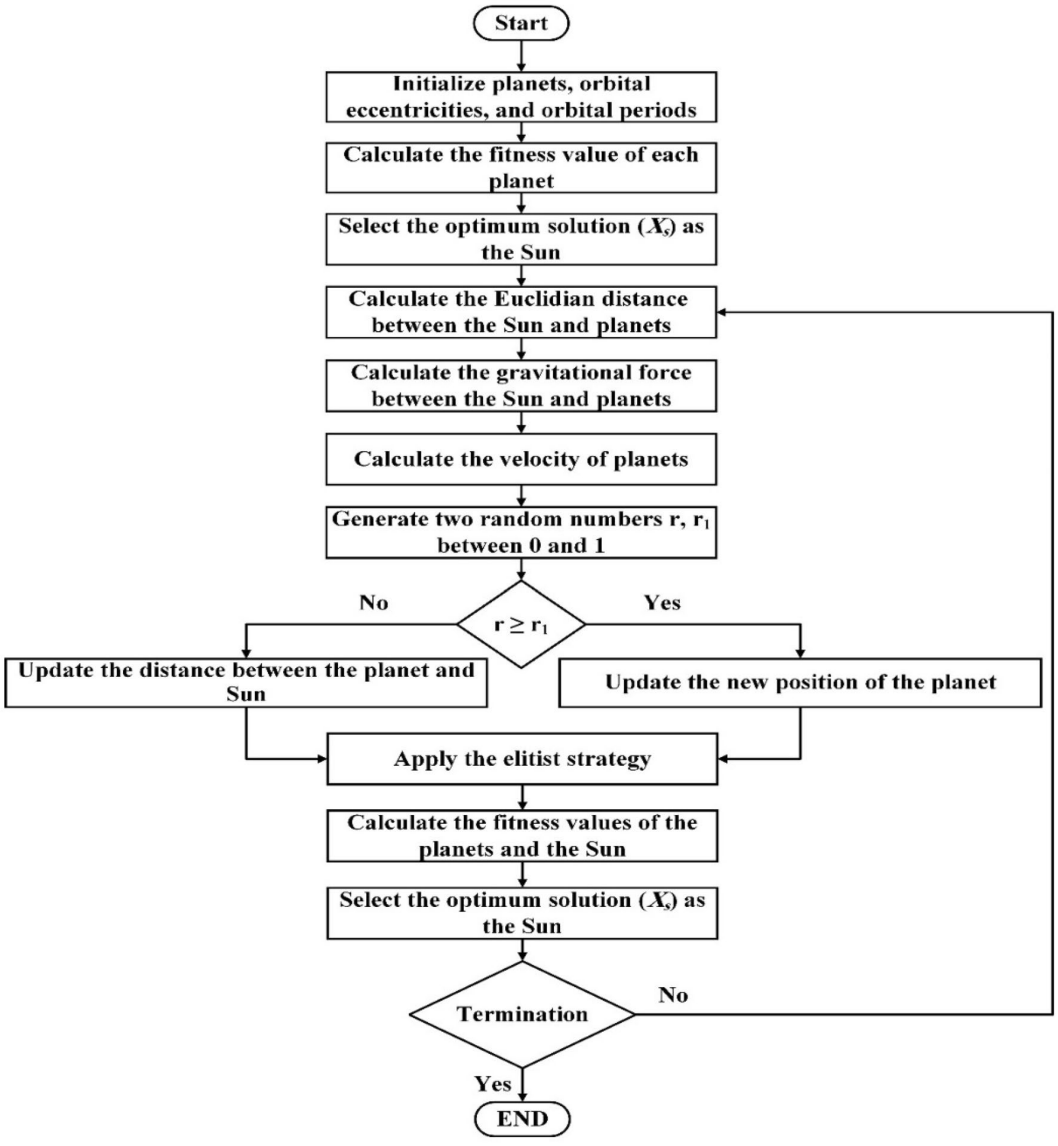
The flowchart of the KOA algorithm.

KOA draws inspiration from Kepler's planetary motion laws, utilizing them for forecasting the planets' positions and velocities at any specific moment^40^. Within the KOA framework, individual planets, characterized by their respective positions, assume the role of candidate solutions. These planetary positions are iteratively adjusted during the optimization process, referencing the best solution achieved thus far (analogous to the Sun in the solar system). The operational steps of the KOA are visually depicted in the provided flow chart, Fig. 2, illustrating the algorithm's computational procedures and its systematic optimization approach.

According to Fig. 2, KOA can be summarized as follows:

#### Initialization process

The initial population of planets will be generated randomly within the defined boundaries of the search space, according to Eq. (30):

30 $${X}_{i}^{j}={rand}_{\left[\mathrm{0,1}\right]}\times \left({X}_{i,up}^{j} - {X}_{i,low}^{j}\right)+ {X}_{i,low}^{j}$$

where ${X}_{i}^{j}$ represents the $i$th planet of $j$th decision variable in the search space. ${X}_{i,up}^{j}$ and ${X}_{i,low}^{j}$ denote the maximum and minimum bounds, respectively.

The orbital $i$th planet eccentricity (${e}_{i}$) is initialized using Eq. (31):

31 $${e}_{i}={rand}_{\left[\mathrm{0,1}\right]}$$

The orbital $i$th planet period (${T}_{i}$) is initialized using Eq. (32):

32 $${T}_{i}=\left|r\right|$$

where $r$ is a random number produced using the normal distribution.

#### Defining the Euclidian distance

The Euclidian distance (${R}_{i}\left(t\right)$) between the Sun ${X}_{s,j}^{t}$ and a planet ${X}_{i,j}^{t}$ is defined as:

33 $${R}_{i}\left(t\right)=\sqrt{\sum_{j=1}^{d}{\left({X}_{s,j}^{t}-{X}_{i,j}^{t}\right)}^{2}}$$

where $d$ stands for the problem dimension.

#### Defining the gravitational force

The gravitational force (${F}_{{g}_{i}}\left(t\right)$) can be calculated using Eq. (34):

34 $${F}_{{g}_{i}}\left(t\right)={e}_{i}\times \mu \left(t\right)\times \frac{\overline{{M }_{s}}\times \overline{{m }_{i}}}{{\overline{{R }_{i}}}^{2}+\varepsilon }+{r}_{1}$$

where $\overline{{M }_{s}}$ and $\overline{{m }_{i}}$ refer to the normalized mass quantities of the Sun and planet, respectively. $\varepsilon$ is a small number. $\mu$ is the constant of universal gravity. ${r}_{1}$ is a random number ranging from 0.0 to 1.0. $\overline{{R }_{i}}$ is the normalized value of ${R}_{i}$.

#### Calculating planets’ velocity

The planet's orbital speed ${V}_{i}^{t}$ as it orbits the Sun is defined as:

35 $${V}_{i}^{t}=\left\{\begin{array}{l}l\times \left(2{r}_{4}{X}_{i}-{X}_{b}\right)+\ddot{\mathcal{l}}\times \left({X}_{a}-{X}_{b}\right)+\left(1-\overline{{R }_{i}}\right)\\ \times \xi \times {U}_{1}\times {r}_{5}\times \left({X}_{i,up}-{X}_{i,low}\right), \overline{{R }_{i}}\le 0.5\\ {r}_{4}\times L\times \left({X}_{a}-{X}_{i}\right)+\left(1-\overline{{R }_{i}}\right)\\ \times \xi \times {U}_{2}\times {r}_{5}\times \left({r}_{3}{X}_{i,up}-{X}_{i,low}\right), Otherwise\end{array}\right.$$

where ${r}_{3}$, ${r}_{4}$, and ${r}_{5}$ are random numbers ranging from 0.0 to 1.0. ${X}_{a}$ and ${X}_{b}$ depict randomly chosen solutions from the population. $\xi$ serves as an indicator to reorient the search.

#### Updating planets’ positions

The new position of each planet is updated using Eq. (36):

36 $${X}_{i}^{t+1}={X}_{i}^{t}+\xi \times {V}_{i}^{t}+\left({F}_{{g}_{i}}^{t}+\left|r\right|\right)\times U\times \left({X}_{s}^{t}-{X}_{i}^{t}\right)$$

#### Updating the distance between the planets and the Sun

The Sun's distance from each planet is updated using Eq. (37):

37 $${X}_{i}^{t+1}={X}_{i}^{t}\times {U}_{1}+\left(1-{U}_{1}\right)\times \left(\frac{{X}_{i}^{t}+{X}_{s}+{X}_{a}^{t}}{3}+h\times \left(\frac{{X}_{i}^{t}+{X}_{s}+{X}_{a}^{t}}{3}-{X}_{b}^{t}\right)\right)$$

where $h$ is a variable used to regulate how far the Sun is from the planet $i$.

#### Elitism

The elitist strategy is described as

38 $${X}_{i,new}^{t+1}=\left\{\begin{array}{ll} {X}_{i}^{t+1}, & f\left({X}_{i}^{t+1}\right)\le f\left({X}_{i}^{t}\right)\\ {X}_{i}^{t} , & Otherwise\end{array}\right.$$

### Nutcracker optimization algorithm (NOA)

**Figure 3:**
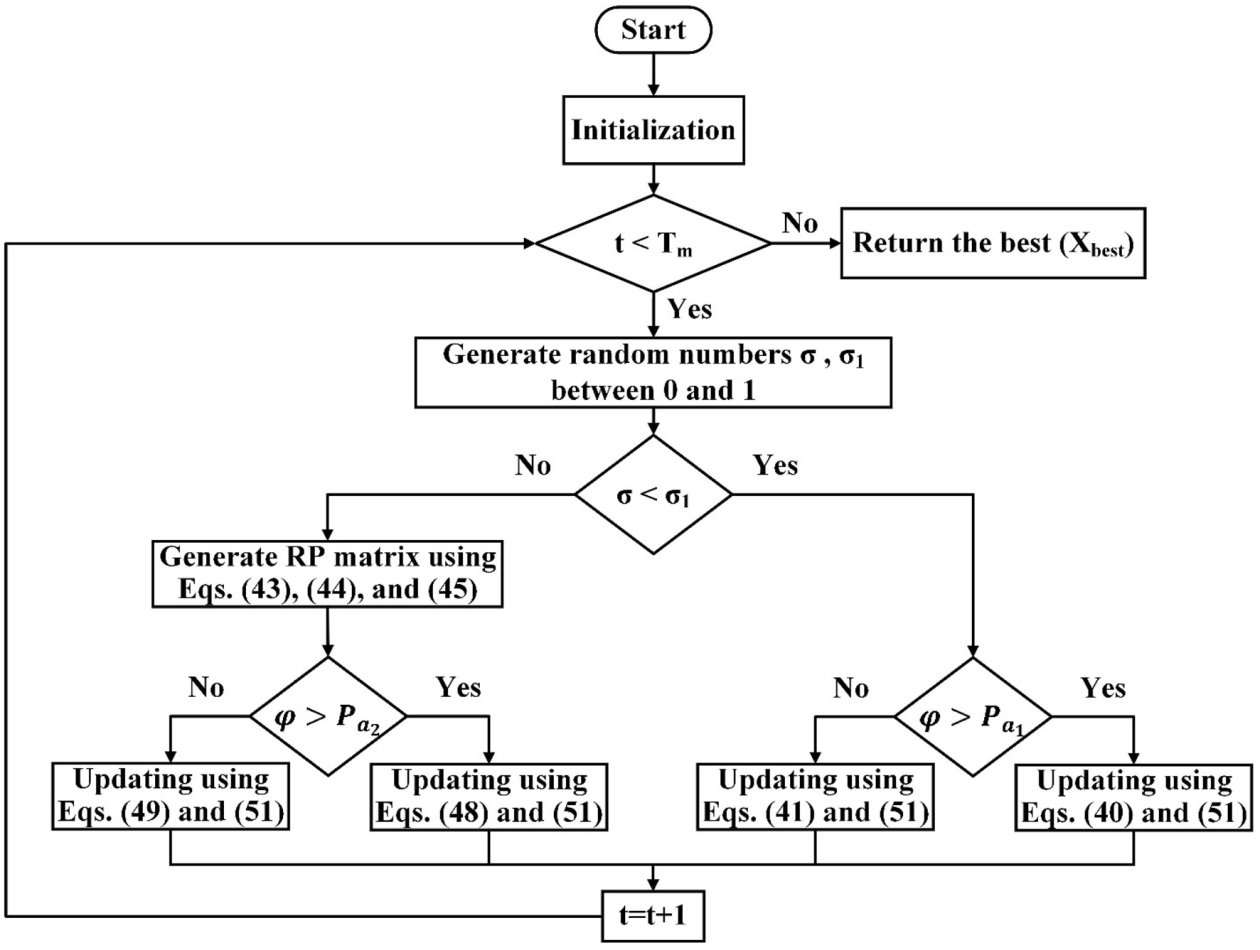
The NOA algorithm flowchart.

NOA models its behavior after Clark's nutcrackers, delineated by two distinct techniques: the foraging and storage technique and the cache-search and recovery technique^41^. The algorithm's operational processes are graphically outlined in the accompanying flow chart, depicted in Fig. 3. This flow chart offers a visual representation of the sequential steps undertaken by the NOA, aligning with the algorithm's emulation of the nutcrackers' natural behaviors.

NOA is summed up in the following steps:

#### Initialization process

The NOA population is initialized by

39 $${X}_{i,j}^{t}=\left({U}_{j} - {L}_{j}\right)\cdot RM + {L}_{j}$$

where ${X}_{i,j}^{t}$ indicates the $i$th nutcracker (feasible solution) of the $j$th decision variable in generation $t$. ${U}_{j}$ and ${L}_{j}$ denote the $j$th decision variable maximum and minimum bounds, respectively. $RM$ represents a random vector ranging from 0.0 to 1.0.

#### Foraging and storage technique

This technique replicates the initial conduct observed in nutcrackers, manifesting during the summer and autumn seasons to gather pine seeds and hoard them. As a result, this technique can be bifurcated into two principal stages: foraging and storage. These stages are expounded upon below:

##### Foraging stage

Every nutcracker starts by examining the cone containing the seeds, as shown in Eq. (40).

40 $${X}_{i}^{t+1}=\left\{\begin{array}{ll}{X}_{i,j}^{t}, & {\tau }_{1}<{\tau }_{2}\\ \left\{\begin{array}{ll}{X}_{m,j}^{t}+\gamma \cdot \left({X}_{A,j}^{t}-{X}_{B,j}^{t}\right)+\mu \cdot \left({r}^{2}\cdot {U}_{j} - {L}_{j}\right), & t\le {T}_{m}/2\\ {X}_{C,j}^{t}+\mu \cdot \left({X}_{A,j}^{t}-{X}_{B,j}^{t}\right)+\mu \cdot \left({r}_{1}<\delta \right)\cdot \left({r}^{2}\cdot {U}_{j} - {L}_{j}\right), & Otherwise\end{array}\right., & Otherwise\end{array}\right.$$

where $\gamma$ is a randomly produced number based on the levy flight. $A$, $B$, and $C$ are three indicators chosen at random from the population. ${\tau }_{1}$, ${\tau }_{2}$, $r$, and ${r}_{1}$ are random numbers ranging from 0.0 to 1.0. ${X}_{m,j}^{t}$ is the average of all solutions in iteration $t$. $\mu$ is a number produced using random numbers ranging from 0 to 1, levy-flight, and normal distribution. $\delta$ is the likelihood of nutcrackers travelling across the entire search space to look for unreachable locations.

##### Storage stage

Nutcrackers start by moving the food acquired in the earlier stage to temporary storage facilities, which can be expressed as follows:

41 $${X}_{i}^{t+1\left(new\right)}=\left\{\begin{array}{lll}{X}_{i}^{t}+\mu \cdot \left({X}_{best}^{t}-{X}_{i}^{t}\right)\cdot \left|\lambda \right|+{r}_{1}\cdot \left({X}_{A}^{t}-{X}_{B}^{t}\right), & {\tau }_{1}<{\tau }_{2}\\ {X}_{best}^{t}+\mu \cdot \left({X}_{A}^{t}-{X}_{B}^{t}\right), & {\tau }_{1}<{\tau }_{3}\\ {X}_{best}^{t}\cdot l, & Otherwise\end{array}\right.$$

where ${X}_{i}^{t+1\left(new\right)}$ denotes a new location in the nutcrackers’ storage region in iteration $t$.${X}_{best}^{t}$ indicates the best solution obtained even now. $\lambda$ is a number produced based on levy flight. $l$ is a linearly decreasing factor from 1.0 to 0.0.

The interchange between the foraging and storage stages is adopted using Eq. (42):

42 $${X}_{i}^{t+1}=\left\{\begin{array}{ll}Eq. (40) , & \varphi >{P}_{{a}_{1}}\\ Eq. (41) , & Otherwise\end{array}\right.$$

where $\varphi$ refers to a random number ranging from 0.0 to 1.0, and ${P}_{{a}_{1}}$ represents a linearly decreasing probability value from 1.0 to 0.0.

#### Cache-search and recovery technique

This technique simulates the second behavior of nutcrackers, which involves looking for and retrieving storage spaces during winter and spring. Therefore, this technique can be bifurcated into two principal stages: cache search and recovery. These stages are expounded upon below:

##### Cache-search stage

The nutcrackers start to identify their caches using a spatial memory methodology. For simplicity, NOA supposes that there are just two Reference Points ($RPs$) (objects) per cache. as shown in Eq. (43).

43 $$RPs=\left[\begin{array}{cc}{RP}_{\mathrm{1,1}}^{t}& {RP}_{\mathrm{1,2}}^{t}\\ \vdots & \vdots \\ {RP}_{i,1}^{t}& {RP}_{i,2}^{t}\\ \vdots & \vdots \\ {RP}_{N,1}^{t}& {RP}_{N,2}^{t}\\ \vdots & \vdots \end{array}\right]$$

where ${RP}_{i,1}^{t}$ and ${RP}_{i,2}^{t}$ represent $RPs$ of $i$th nutcracker cache position in generation $t$.

The ${RP}_{i,1}^{t}$ and ${RP}_{i,2}^{t}$ are described as follows:

44 $${RP}_{i,1}^{t}=\left\{\begin{array}{ll}{X}_{i}^{t}+\alpha \cdot {\text{cos}}\left(\theta \right)\cdot \left({X}_{A}^{t}-{X}_{B}^{t}\right)+\alpha \cdot RP, & \theta =\pi /2\\ {X}_{i}^{t}+\alpha \cdot {\text{cos}}\left(\theta \right)\cdot \left({X}_{A}^{t}-{X}_{B}^{t}\right), & Otherwise\end{array}\right.$$

45 $${RP}_{i,2}^{t}=\left\{\begin{array}{ll}{X}_{i}^{t}+\left(\alpha \cdot {\text{cos}}\left(\theta \right)\cdot \left(\left(U-L\right)\cdot {\tau }_{3}+L\right)+\alpha \cdot RP\right)\cdot {U}_{2}, & \theta =\pi /2\\ {X}_{i}^{t}+\alpha \cdot {\text{cos}}\left(\theta \right)\cdot \left(\left(U-L\right)\cdot {\tau }_{3}+L\right)\cdot {U}_{2}, & Otherwise\end{array}\right.$$

where $\alpha$ linearly decreases from one to zero. ${X}_{A}^{t}$ is the $A$th nutcracker cache position in iteration $t$. $\theta$ stands for the nutcracker angle of view, which is randomly selected between 0 and π. $RP$ is a random position.

Nutcracker's new position can be updated via ${RP}_{i,1}^{t}$:

46 $${X}_{i}^{t+1}=\left\{\begin{array}{ll}{X}_{i}^{t}, & f\left({X}_{i}^{t}\right)<f\left({RP}_{i,1}^{t}\right)\\ {RP}_{i,1}^{t}, & Otherwise\end{array}\right.$$

If the nutcracker is unable to recall where he buried his food utilizing ${RP}_{i,1}^{t}$, ${RP}_{i,2}^{t}$ will be used. Therefore, ${RP}_{i,2}^{t}$ is used to update Nutcracker spatial memory via Eq. (47):

47 $${X}_{i}^{t+1}=\left\{\begin{array}{ll}{X}_{i}^{t}, & f\left({X}_{i}^{t}\right)<f\left({RP}_{i,2}^{t}\right)\\ {RP}_{i,2}^{t}, & Otherwise\end{array}\right.$$

The exchange between ${RP}_{i,1}^{t}$ and ${RP}_{i,2}^{t}$ is achieved as follows:

48 $${X}_{i}^{t+1}=\left\{\begin{array}{ll}Eq. (46) , & f\left({\text{Eq}}. (46) \right)<f\left({\text{Eq}}. (47) \right)\\ Eq. (47) , & Otherwise\end{array}\right.$$

##### Recovery stage

When the nutcracker can recall the cache location using either ${RP}_{i,1}^{t}$ or ${RP}_{i,2}^{t}$, there are two outcomes for each: either there is food or there isn't. This process can be expressed as follows:

49 $$X_{i}^{{t + 1}} = \left\{ {\begin{array}{*{20}l} {\left\{ {\begin{array}{*{20}l} {X_{{i,j}}^{t} ,} & {\tau _{3} < \tau _{4} } \\ {X_{{i,j}}^{t} + r_{1} \cdot \left( {X_{{best,j}}^{t} - X_{{i,j}}^{t} } \right) + r_{2} \cdot \left( {RP_{{i,1}}^{t} - X_{{C,j}}^{t} } \right),} & {Otherwise} \\ \end{array} } \right.,} \hfill & {\tau _{7} < \tau _{8} } \hfill \\ {\left\{ {\begin{array}{*{20}l} {X_{{i,j}}^{t} ,} \hfill & {\tau _{5} < \tau _{6} } \hfill \\ {X_{{i,j}}^{t} + r_{1} \cdot \left( {X_{{best,j}}^{t} - X_{{i,j}}^{t} } \right) + r_{2} \cdot \left( {RP_{{i,2}}^{t} - X_{{C,j}}^{t} } \right),} \hfill & {Otherwise} \hfill \\ \end{array} } \right.,} \hfill & {Otherwise} \hfill \\ \end{array} } \right.$$

where ${\tau }_{3}$, ${\tau }_{4}$, ${\tau }_{5}$, ${\tau }_{6}$, ${\tau }_{7}$, and ${\tau }_{8}$ are random numbers ranging from 0.0 to 1.0.

The interchange between the cache-search and recovery stages is adopted using Eq. (50):

50 $${X}_{i}^{t+1}=\left\{\begin{array}{ll}Eq. (48) , & \varphi >{P}_{{a}_{2}}\\ Eq. (49) , & Otherwise\end{array}\right.$$

where ${P}_{{a}_{2}}$ corresponds to a probability value of 0.2.

Nutcracker's current position can be enhanced as follows:

51 $${X}_{i}^{t+1}=\left\{\begin{array}{ll}{X}_{i}^{t+1}, & f\left({X}_{i}^{t+1}\right)<f\left({X}_{i}^{t}\right)\\ {X}_{i}^{t} , & Otherwise\end{array}\right.$$

### Dandelion algorithm

**Figure 4:**
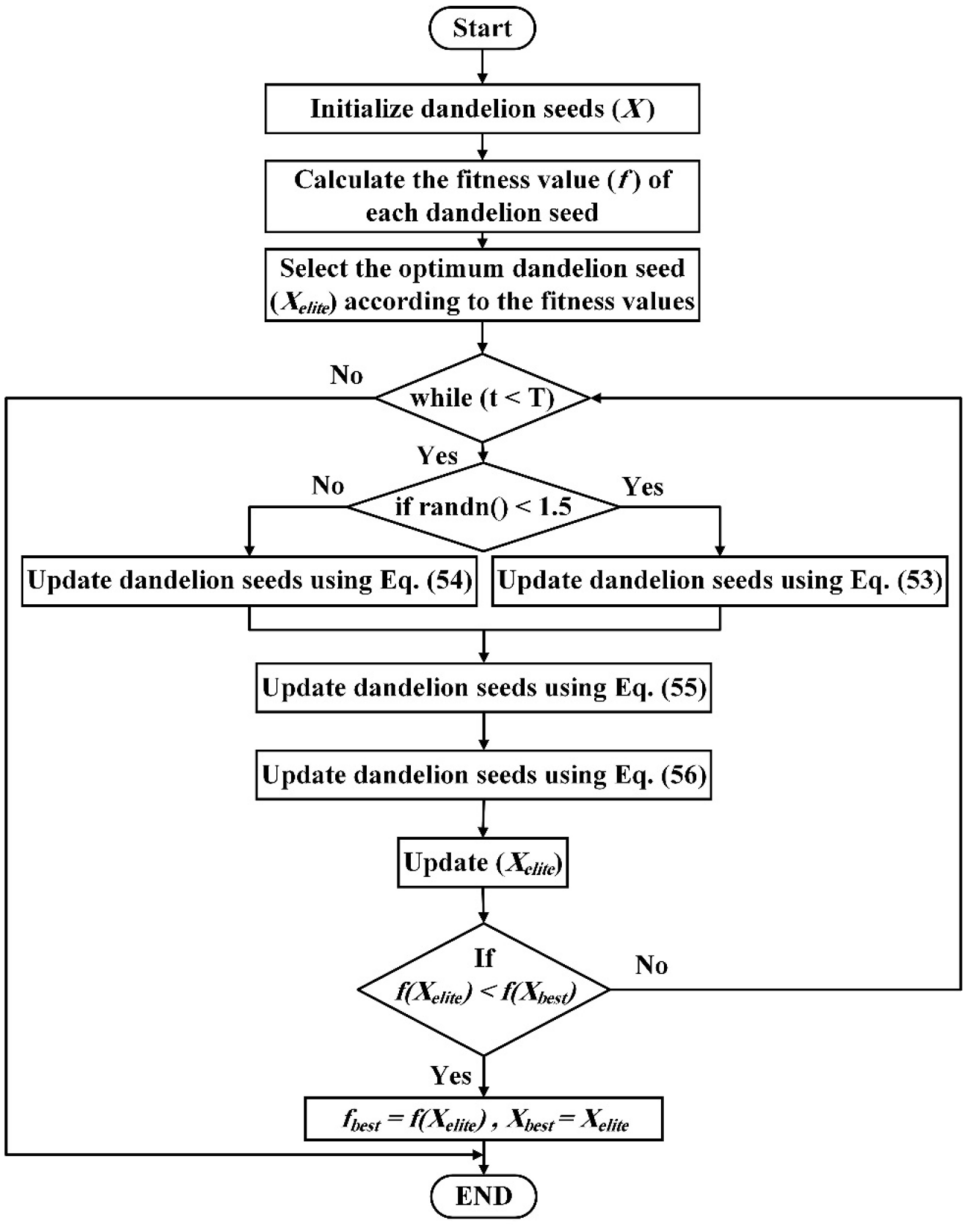
The suggested DA algorithm flowchart.

The Dandelion Algorithm (DA) emulates the prolonged aerial voyage of dandelion seeds, a journey characterized by three distinct phases: ascent, descent, and settlement^42^. The computational procedures of the algorithm are graphically depicted in the accompanying flowchart, Fig. 4, serving as an illustrative overview of the DA's operational process.

DA is summed up in the following steps.

#### Initialization and configuration of algorithm parameters

The initial population of dandelion seeds is generated using the following equation:

52 $${X}_{i}=rand \times (UB - LB) + LB$$

where ${X}_{i}$ indicates the $i$th dandelion seed (feasible solution). $rand$ represents a random value between [0, 1]. $LB$ and $UB$ represent the decision variables’ maximum and minimum bounds, respectively.

#### Ascending phase

Dandelion seeds disperse from their parent plant once they attain a suitable height. This stage comprises two potential scenarios for the movement of dandelion seeds, which are contingent on factors such as wind speed, air resistance, and humidity.

##### Case 1

With increasing wind strength, the dandelion achieves greater height and scatters its seeds over a wider range. This scenario can be mathematically represented as follows:

53 $${X}_{t+1}={X}_{t}+rand*\left(\frac{{t}^{2}}{{T}^{2}}-\frac{2t}{T}+1\right)*\frac{1}{{e}^{2\theta }}*\frac{sin\left(2\theta \right)}{2}*ln Y*\left({X}_{s} - {X}_{t}\right)$$

where ${X}_{t}$ represents the position of the seed at iteration t. ${X}_{t+1}$ denotes the seed position at iteration t + 1. ${X}_{s}$ signifies a random location within the search space. The term $ln Y$ represents the logarithmic normal distribution. $\theta$ represents a random number falling within the range of [− π, π]. $T$ represents the maximum number of iterations.

##### Case 2

Under rainy conditions, dandelion seeds encounter challenges in achieving optimal ascent against the wind. This situation can be expressed mathematically as follows:

54 $${X}_{t+1}={X}_{t}*\left(1-rand*\left(\frac{{T}^{2}-2T+{t}^{2}-2t+2}{{T}^{2}-2T+1}\right)\right)$$

#### Descent phase

This phase promotes the exploration process and can be mathematically represented as follows:

55 $${X}_{t+1}={X}_{t}-rand*\left(\frac{{t}^{2}}{{T}^{2}}-\frac{2t}{T}+1\right)*{\beta }_{t}*\left(\left(\frac{1}{ pop }\sum_{i=1}^{pop}{X}_{i}\right)-rand*\left(\frac{{t}^{2}}{{T}^{2}}-\frac{2t}{T}+1\right)*{\beta }_{t}*{X}_{t}\right)$$

where ${\beta }_{t}$ represents Brownian motion and is a random number drawn from a normal distribution. $pop$ is the population size.

#### Landing phase

The landing phase facilitates the exploitation process and can be defined by the following equation:

56 $${X}_{t+1} = {X}_{elite}+\frac{s*w*\Gamma \left(1+\beta \right)*{\text{sin}}\left(\frac{\pi \beta }{2}\right)}{{\left|t\right|}^{\frac{1}{\beta }}*\Gamma \left(\frac{1+\beta }{2}\right)*\beta *{2}^{\left(\frac{\beta -1}{2}\right)}}*rand*\left(\frac{{t}^{2}}{{T}^{2}}-\frac{2t}{T}+1\right)*\left({X}_{elite}-\frac{2t}{T}*{X}_{t}\right)$$

where ${X}_{elite}$ indicates the seed's optimal position during ${i}{\text{th}}$ iteration. $s$ set at a fixed value of 0.01. $w$ and $t$ are random numbers within the range of [0, 1]. $\beta$ is fixed at 1.5.

#### Termination

The DA algorithm concludes its execution and provides the optimal solution vector after a certain number of iterations (T).

## Results and discussion

**Figure 5:**
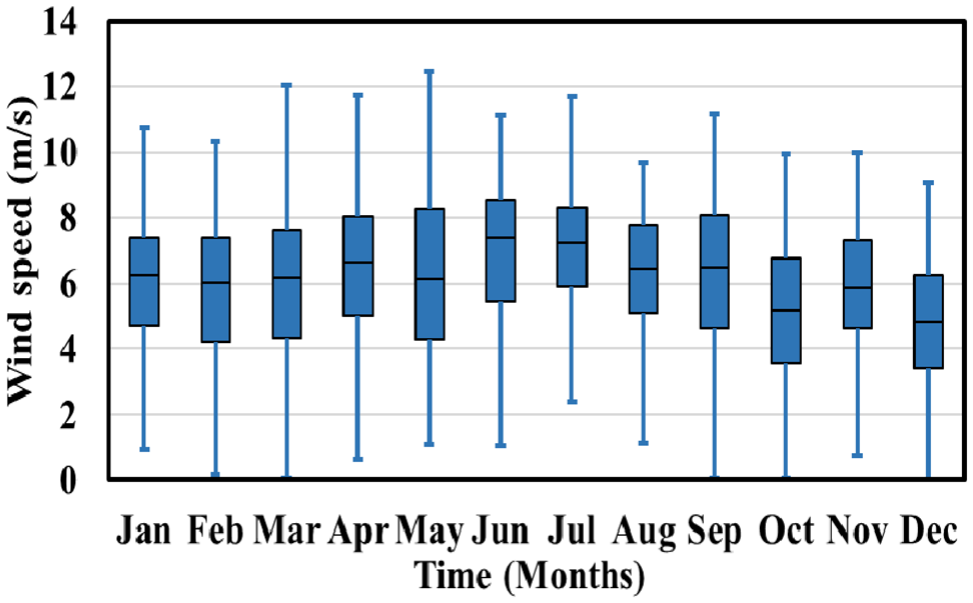
Monthly wind speed.

**Figure 6:**
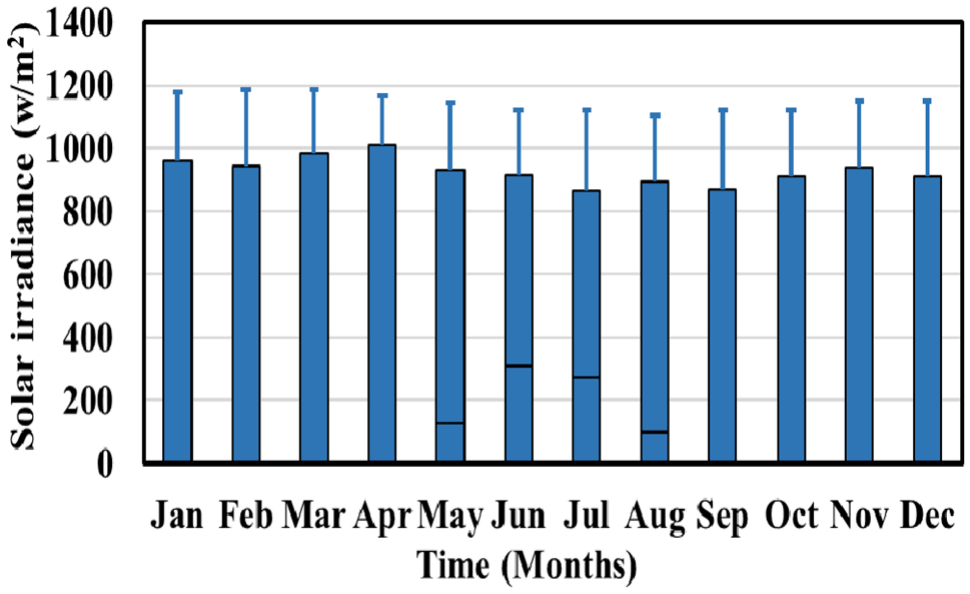
Monthly solar irradiance.

The microgrid model proposed in this study is situated in the city of Benban, located within the Aswan Governorate. Geographically, Benban is positioned at a longitude of 32.870°E and a latitude of 24.440°N. Figure 5 displays the consistent fluctuation of wind speed throughout this period, reaching its peak value of 12.4470 m/s at 6 p.m.^43^. This value then gradually diminishes to reach zero by 5 p.m., remaining at this level until 6 a.m. the following day^44^. In contrast, Fig. 6 illustrates a gradual and nonlinear rise in solar irradiance throughout the day, starting from zero at 6 a.m. and peaking at noon with a maximum recorded value of 1187.59 W/m^2^.

**Figure 7:**
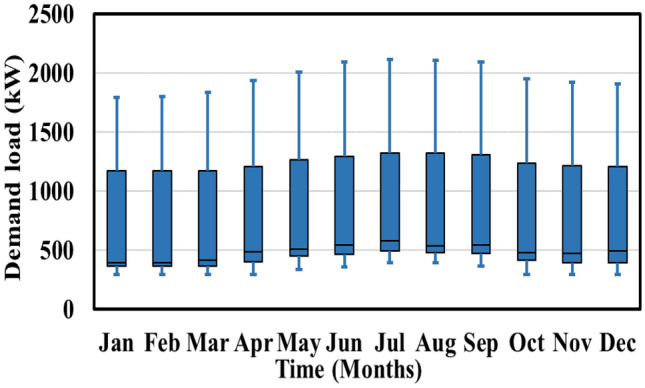
Monthly demand profile.

The associated costs for electricity transactions in this microgrid scenario are as follows: purchasing electricity from the utility grid costs 0.20 $/kWh from midnight to 8 a.m., 0.50 $/kWh from 8 a.m. to 4 p.m., and 0.30 $/kWh from 4 p.m. to midnight, while selling electricity back to the utility is priced at 0.06685 $/kWh^45^. Additionally, Fig. 7 displays the monthly distribution of load demand throughout the year, indicating a peak demand of 2115.40 kW at 5:00 p.m. and a minimum demand of 290.0 kW at 10:00 a.m.^46^. The initial price of electricity remains constant at 0.5 $/kWh within each respective period.

To evaluate the effectiveness of the proposed optimization technique, a comparative analysis of performance is conducted. Four distinct operational scenarios (each corresponding to different optimization techniques) are explored for the microgrid model incorporating RGDP DR. This investigation aims to elucidate the impact of the recommended optimization strategy.

**Figure 8:**
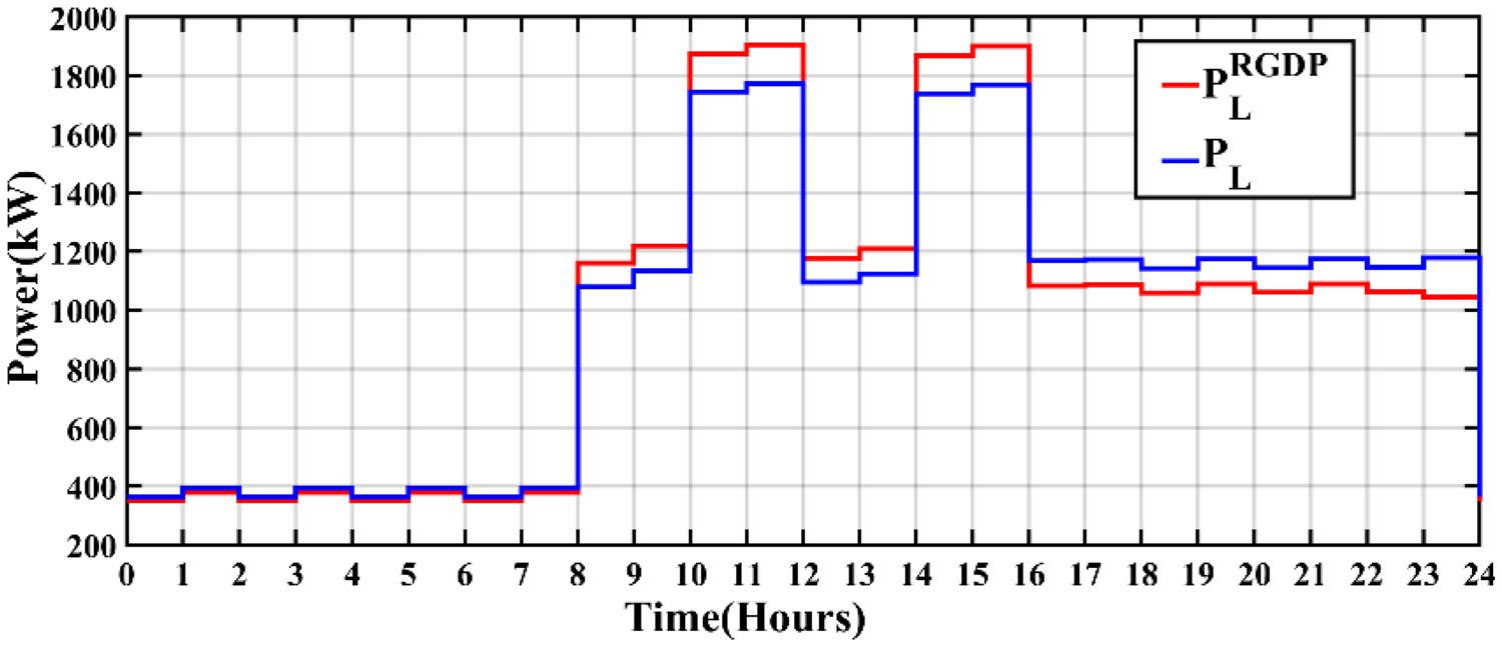
RGDP DR demand profile.

**Figure 9:**
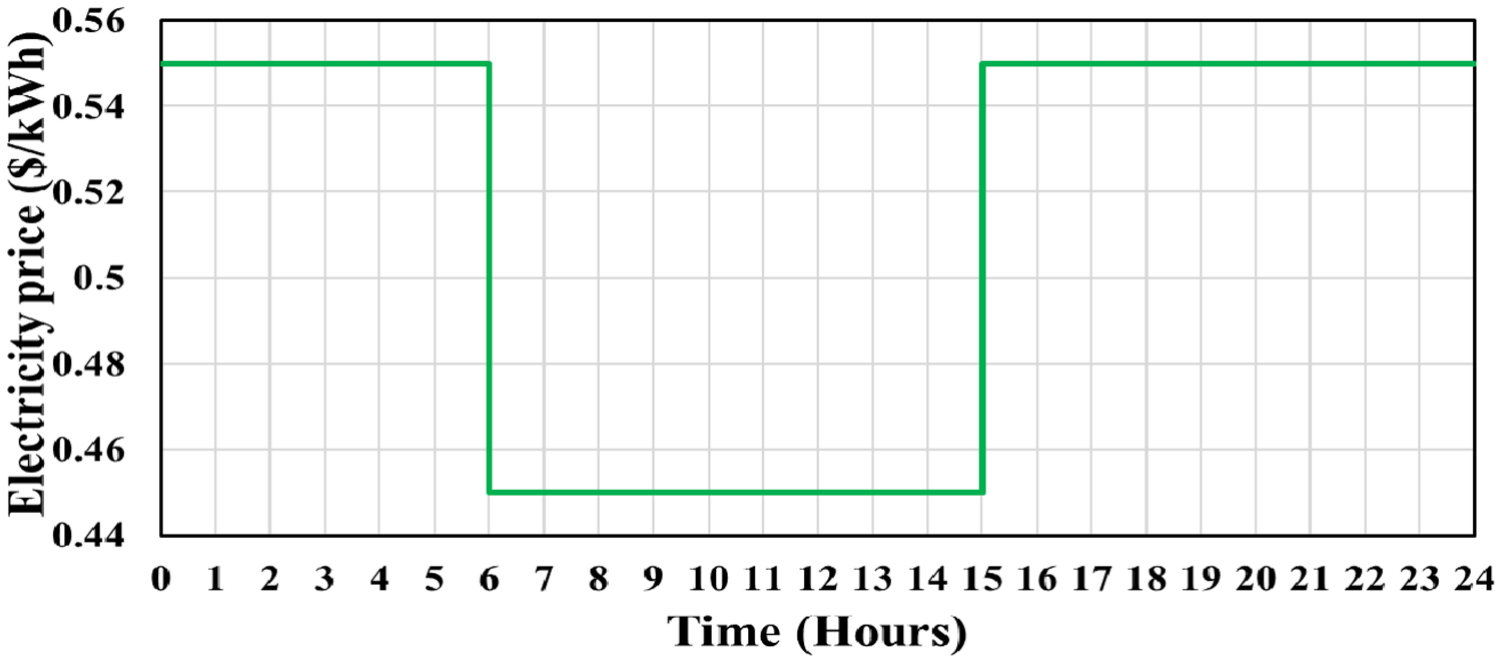
The generated electricity cost with RGDP DR deployment.

Across all scenarios, crucial metrics including energy consumption, decrease in energy consumption, incentives, and penalties result in values of 707,959 kWh, zero, zero, and zero, respectively. The pricing framework encompasses maximum and minimum boundaries set at 0.550 and 0.450 $/kWh, respectively. Figure 8 illustrates the transformation in economic load demand profiles, both before and after the implementation of RGDP DR. Furthermore, the alteration in electricity pricing resulting from RGDP DR is visually presented in Fig. 9, calculated using Eq. (28). The results of implementing optimization techniques for obtaining the optimal size of RES for the microgrid will be discussed in the following subsections.

### Scenario 1: DA algorithm

**Figure 10:**
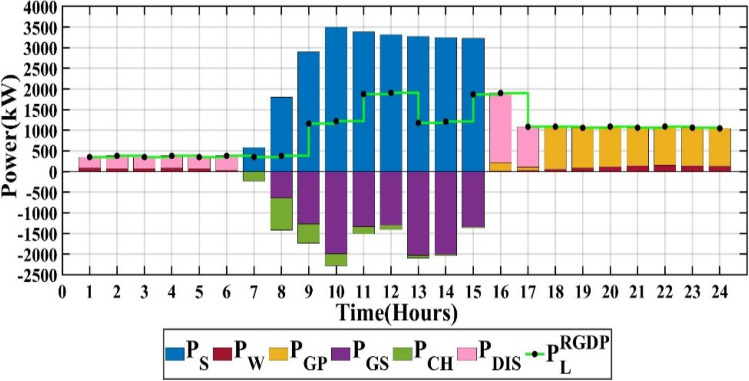
Scenario 1 of MG.

The proposed microgrid configuration entails capacities for PV, wind, battery, and converter set at 6579 kW, 937 kW, 3482 kW, and 3212 kW, respectively. These designations align with the life cycle emissions of 2,696,972 kgCO_2_-eq/Year, the total microgrid cost of 1,246,864 $/Year, and the customer bill totaling 3,845,911 $/Year, as elucidated within Table 2. The hourly output power from each Renewable Energy Source (RES) throughout 24 h is visually illustrated in Fig. 10, employing the Dandelion Algorithm (DA).

**Table 2:** Overall comparison of the studied scenarios.

	DA	PSO	NOA	KOA
PV capacity (kW)	6579	6566	6542	6551
Wind capacity (kW)	937	926	924	908
Battery capacity (kW)	3482	3523	3537	3617
Converter capacity (kW)	3212	3211	3192	3241
Life cycle emissions (LCE) (kgCO_2_-eq/yr)	2,696,972	2,694,292	2,690,314	2,683,489
Emission cost ($/year)	53,940	53,886	53,807	53,670
Components cost ($/year)	1,192,924	1,192,980	1,193,064	1,193,235
Total cost of microgrid ($/year)	1,246,864	1,246,866	1,246,871	1,246,905
Customer bill ($/year)	3,845,911	3,846,043	3,846,108	3,846,235

### Scenario 2: PSO algorithm

**Figure 11:**
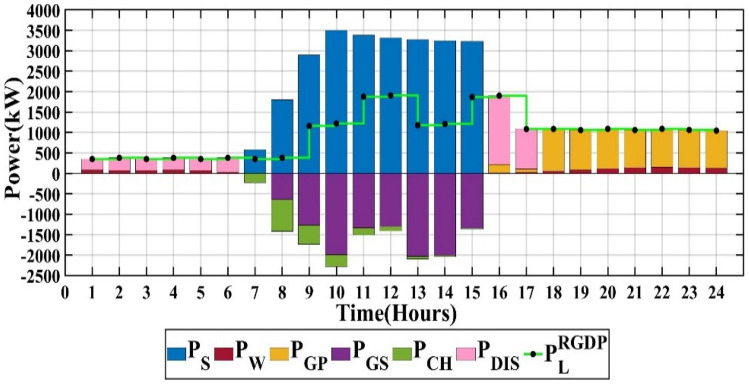
Scenario 2 of MG.

The capacities of PV, wind, battery, and converter are 6566 kW, 926 kW, 3523 kW, and 3211 kW, respectively. The life cycle emissions amount to 2,694,292 kgCO_2_-eq/Year, the total microgrid cost is 1,246,866 $/Year, and the customer bill reaches 3,846,043 $/Year, as detailed in Table 2. The output power of each RES for each hour over a single day is depicted in Fig. 11 through the application of PSO.

### Scenario 3: NOA algorithm

**Figure 12:**
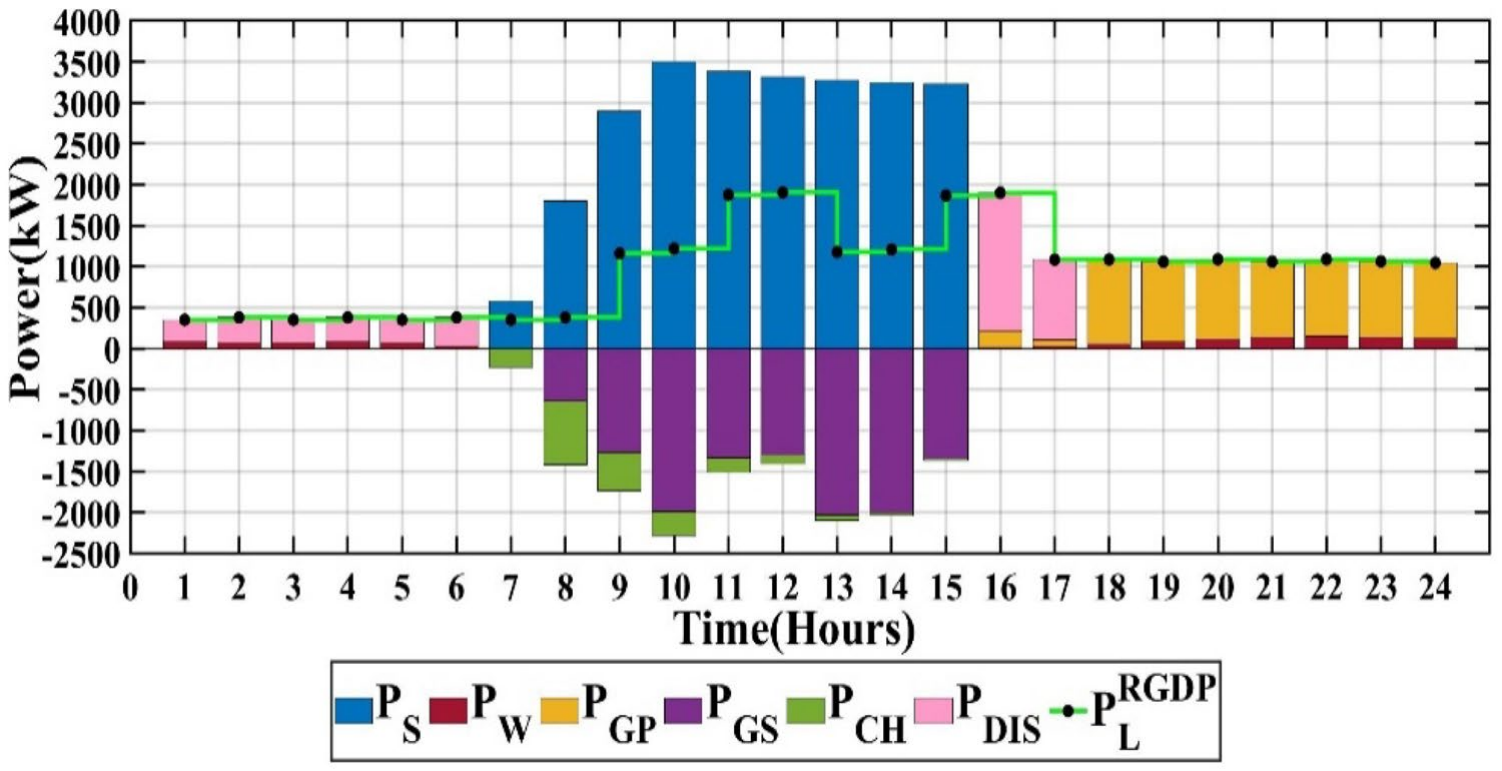
Scenario 3 of MG.

The capacities of PV, wind, battery, and converter are 6542 kW, 924 kW, 3537 kW, and 3192 kW, respectively. The life cycle emissions amount to 2,690,314 kgCO_2_-eq/Year, the total microgrid cost is 1,246,871 $/Year, and the customer bill reaches 3,846,108 $/Year, as detailed in Table 2. The output power of each RES for each hour over a single day is illustrated in Fig. 12, utilizing the NOA.

### Scenario 4: KOA algorithm

**Figure 13:**
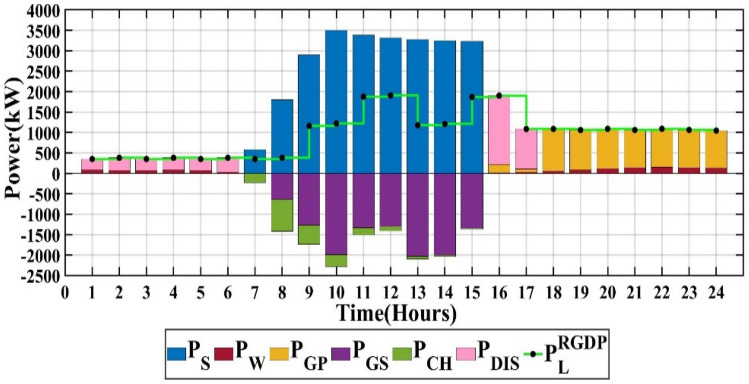
Scenario 4 of MG.

The capacities of PV, wind, battery, and converter are 6551 kW, 908 kW, 3617 kW, and 3241 kW, respectively. The life cycle emissions amount to 2,683,489 kgCO_2_-eq/Year, the total microgrid cost is 1,246,905 $/Year, and the customer bill reaches 3,846,235 $/Year, as detailed in Table 2. The output power of each Renewable Energy Source (RES) for each hour over a single day is depicted in Fig. 13 using the KOA.

## Conclusion

This paper introduces an innovative methodology for determining the optimal size of a grid-connected microgrid (MG) through an energy management framework with two primary objectives: minimizing the total annual cost and reducing life cycle emissions. The devised microgrid architecture incorporates distributed energy resources such as Battery Energy Storage Systems (BESS), wind turbines (WT), and photovoltaics (PV). A comprehensive mathematical model is presented, integrating the RGDP-DR approach to ascertain the optimal grid-connected MG size. The RGDP DR strategy is devised to address the disparities between demand load and renewable energy source (RES) generation.

The study conducts a thorough comparative analysis involving four optimization techniques: Dandelion Algorithm (DA), Particle Swarm Optimization (PSO), Nature-Inspired Optimization Algorithm (NOA), and Knowledge Optimization Algorithm (KOA). The evaluation metrics encompass life cycle emissions, the optimal microgrid cost, and customer billing. Simulation results demonstrate the superiority of the proposed DA in achieving the lowest microgrid cost and customer bill, outperforming the other optimization methods. Importantly, this evaluation considers only 10% of the load in the management strategy.

DA demonstrates a minimal total annual cost of $1,246,864, leading to a marginal difference compared to other techniques. PSO closely follows, with a total annual cost of $1,246,866, showing comparable performance to DA. NOA and KOA yield similar results, with total annual costs of $1,246,871 and $1,246,905, respectively. In terms of the annual customer bill, DA results in the lowest at $3,845,911, showcasing a distinct advantage over other optimization methods. PSO closely trails DA, with an annual customer bill of $3,846,043, indicating competitive performance. NOA and KOA exhibit comparable annual customer bills, standing at $3,846,108 and $3,846,235, respectively.

In conclusion, this research establishes that the proposed framework offers an optimal approach for developing a sustainable microgrid driven by renewable energy sources. The numerical evidence supports the claim of the Dandelion Algorithm's effectiveness, particularly in minimizing both microgrid cost and customer billing, even when considering only 10% of the load in the management strategy.

In future research, the proposed RGDP-based DR may be employed on interconnected multi-nano grids and microgrids to find the optimal configuration and size of these interconnected microgrids, considering MG configurations, load types, and uncertainties.

## Data Availability

The datasets generated during the current study are available from the corresponding author upon reasonable request.

## List of symbols

$${N}_{S}$$: The number of PV modules
$${F}_{S}$$: PV reduction factor
$${P}_{L}^{Z}\left(t\right)$$: $${z}{\text{th}}$$ Scenario load power (kW)
$${\rho }_{o}\left(i\right)$$: The initial electricity price ($)
$${P}_{L}\left(i\right)$$: The initial load demand (kW)
$$E\left(i,i\right)$$: Self-elasticity
$$E\left(i,j\right)$$: Cross-elasticity
$${\rho }_{rgdp}\left(i\right)$$: The RGDP electricity price ($/kWh).
$${P}_{L}^{RGDP}\left(i\right)$$: The final economic RGDP load model (kWh)
$${e}_{i}$$: The orbital eccentricity for the $$i$$ th planet.
$${R}_{i}\left(t\right)$$: The Euclidian distance
$${V}_{i}\left(t\right)$$: The $$i$$ th planet velocity.
$$\varphi$$: A random number between zero and one.
$$\lambda$$: A number produced based on levy flight.
$$\xi$$: An indicator to reorient the search.
$$h$$: A variable used to regulate how far the Sun is from the planet $$i$$.
$$SOC\left(t\right)$$: Battery state of charge (kWh)
$${{\text{E}}}_{pen}$$: Emissions penalty ($/ton)
$${RPC}_{AN}$$: Annual replacement cost
$${OMC}_{AN}$$: Annual operating and maintenance cost
$${D}_{r}$$: Real discount rate (%)
$${N}_{k}$$: $${k}{\text{th}}$$ Component number of units
$${P}_{R\_{\text{k}}}$$: $${k}{\text{th}}$$ Component rated capacity (kW)
$${C}_{i\_k}$$: $${k}{\text{th}}$$ Component initial cost ($/kW)
$${NR}_{k}$$: $${k}{\text{th}}$$ Component number of replacements
$${L}_{k}$$: $${k}{\text{th}}$$ Component lifetime (years)
$${F}_{k}$$: $${k}{\text{th}}$$ Component emissions (kgCO_2_-eq/kWh)
$${{\text{E}}}_{{\text{k}}}$$: $${k}{\text{th}}$$ Component produced energy (kWh)
$${X}_{elite}$$: The dandelion seed's best position
$${T}_{i}$$: The $$i$$ th planet orbital period
$${F}_{{g}_{i}}\left(t\right)$$: The attraction force
$$\mu$$: A number produced using random numbers ranging from 0 to 1, levy-flight, and normal distribution.
$$l$$: A linearly decreasing factor from 1.0 to 0.0.
$$\delta$$: The likelihood of nutcrackers travelling across the entire search space to look for unreachable locations
